# Supplementary material for: Prevalence and risk factors of hearing loss in the Chinese population aged 45 years and older: Findings from the CHARLS baseline survey
Source: PLoS One. 2024 Sep 23;19(9):e0310953. doi: 10.1371/journal.pone.0310953 (PMC11419390; doi:10.1371/journal.pone.0310953)
Supplement: Attachment — Submitted filename: PLOSONE Review.docx [file pone.0310953.s001.docx]

Dear Author,

Thank you for submitting this paper to the PLOSONE journal.

-I have a question about the criteria to classify hearing impairment: reporting hearing problem, using a HA and having a poor hearing status (how this was evaluated?)

- What some of the suggestion to help those who are at higher risk to develop HL?

-what is your suggestions to reduce the prevalence of HL among the targeted population?

- What are the limitations to the study?
