# Supplementary material for: Prevalence and risk factors of hearing loss in the Chinese population aged 45 years and older: Findings from the CHARLS baseline survey
Source: PLoS One. 2024 Sep 23;19(9):e0310953. doi: 10.1371/journal.pone.0310953 (PMC11419390; doi:10.1371/journal.pone.0310953)
Supplement: Attachment — Submitted filename: Response to Reviewers.docx [file pone.0310953.s002.docx]

Dear Academic Editor,

Thank you for your thorough review and constructive feedback. Below are the streamlined responses to each of your inquiries:

**Style and File Naming Requirements:**

We have meticulously reviewed and adjusted our manuscript to comply with PLOS ONE's formatting and file naming guidelines, utilizing the provided LaTeX templates.

**Data Availability Statement:**

We confirm that the "minimal data set" necessary to replicate our study's findings is available in Dryad with the DOI: https://doi.org/10.5061/dryad.mpg4f4r85, and there are no restrictions on data sharing.

**Ethics Statement Placement:**

The ethics statement has been relocated to the Methods section of the manuscript, ensuring it is included appropriately for publication.

**Copyright Concerns for Map Images:**

In response to the copyright issue with Figure 2, we have replaced the copyrighted map images with a bar chart that is free from any copyright restrictions.

We trust that these revisions have adequately addressed your concerns and have further strengthened the quality of our submission. We look forward to your continued guidance and feedback.

Sincerely,

Deping Sun

2024-6-28

Dear Reviewer #1,

Thank you for your insightful comments and questions on our manuscript submitted to PLOS ONE. We have taken your feedback into careful consideration and have made the following revisions and clarifications in our manuscript:

**Criteria for Classifying Hearing Impairment:**

We have clarified our methodology for classifying hearing impairment using a validated self-administered questionnaire. The two key questions regarding the use of hearing aids and self-assessed hearing quality on a Likert scale have been detailed in the revised manuscript to ensure transparency and replicability.

**Suggestions for Those at Higher Risk of Developing Hearing Loss (HL):**

We have expanded the discussion section to include several strategies aimed at assisting individuals at higher risk of developing HL. These include routine hearing screenings, educational programs, gender-neutral access to resources, community outreach, workplace protection, social engagement, and targeted initiatives for ethnic minorities.

**Strategies to Reduce the Prevalence of HL Among the Targeted Population:**

The same recommendations listed above are proposed as strategies to reduce the prevalence of HL. These have been concisely summarized and integrated into the discussion to offer a comprehensive approach to prevention.

**Limitations of the Study:**

We have acknowledged and concisely summarized the key limitations of our study, including the cross-sectional design, reliance on self-reported data, potential lack of regional representation, absence of audiometric testing, and possible residual confounding factors. These limitations are discussed to provide a balanced view of our research findings and to highlight areas for improvement in future studies.

We trust that these revisions adequately address your questions and concerns, and we are grateful for the opportunity to enhance our manuscript based on your feedback.

Sincerely,

Deping Sun

2024-6-28

Dear Reviewer #2,

Thank you for your detailed review and constructive feedback on our manuscript, “Prevalence and Risk Factors of Hearing Loss in the Chinese Population Aged 45 and Older: Findings from the CHARLS Baseline Survey.” We have taken your suggestions seriously and have made the following revisions:

**Introduction Enhancement:**

We have expanded the introduction to discuss the significance of self-reported hearing loss surveys in large-scale studies and have clarified our study’s aims and objectives at the end of the section. Additionally, we have improved referencing to support our statements.

**Exclusion Criteria Explanation:**

We have provided a clear explanation of why participants with missing weight data were excluded, emphasizing its importance for the representativeness and accuracy of our findings.

**Questionnaire Description:**

We have revised the manuscript to confirm that the full, validated questionnaire was used, maintaining consistency with previous research.

**Hearing Impairment Classification Clarification:**

We have succinctly clarified the classification criteria for hearing impairment in the manuscript, ensuring that the distinctions between categories are clear to the reader.

**Terminology and Table Revisions:**

Table 1 and the manuscript text have been revised for enhanced clarity and terminology. Gender terms have been standardized, and abbreviations have been defined in the text.

**Results Based on Hearing Impairment Classification:**

We have included an analysis of the low prevalence of hearing aid use in our discussion, suggesting potential underdiagnosis and barriers to adoption.

We believe these revisions address your concerns and have significantly improved the quality and clarity of our manuscript. We appreciate the opportunity to refine our work based on your comments.

Sincerely,

Deping Sun

2024-6-28
