# Supplementary material for: Prevalence and risk factors of hearing loss in the Chinese population aged 45 years and older: Findings from the CHARLS baseline survey
Source: PLoS One. 2024 Sep 23;19(9):e0310953. doi: 10.1371/journal.pone.0310953 (PMC11419390; doi:10.1371/journal.pone.0310953)
Supplement: Attachment — Submitted filename: Response to Reviewers -second.docx [file pone.0310953.s003.docx]

Dear Academic Editor,

We would like to express our gratitude for the opportunity to revise our manuscript entitled "[Manuscript Title]" and for the constructive comments provided by the reviewers. We have taken the feedback seriously and have made the following revisions to our manuscript:

**Financial Disclosure Statement:** We have included an updated financial disclosure statement in our cover letter as requested, although no changes were necessary.

**Figure File Resubmission:** We have uploaded our figure files to the Preflight Analysis and Conversion Engine (PACE) and have incorporated the recommended modifications. Specifically, we have replaced Figure 1 and Figure 2 with the revised versions provided by PACE.

**Laboratory Protocols:** We have deposited our laboratory protocols on protocols.io to enhance the reproducibility of our results, as suggested. The protocols have been assigned their own identifiers (DOIs) for independent citation in the future. We have included the DOI link (https://doi.org/10.5061/dryad.mpg4f4r85) in our methods section, following the guidelines provided.

**Reference List:** We have meticulously reviewed and updated our reference list to ensure it is complete and accurate. We have confirmed that there are no citations of retracted articles in our manuscript. We have adhered to the "PloS" style for reference formatting and have made sure that all references are up to date.

**Citation of Validation Studies:** In response to the additional editor comments, we have now provided citations for the validation studies of the questionnaire used in our research. Three relevant studies have been added to the manuscript, ensuring that our use of the validated questionnaire is appropriately referenced.

Thank you again for your guidance. We look forward to the possibility of publication in PLOS ONE.

Sincerely,

Deping Sun

2024-8-17
